# SamplEase: a simple application for collection and organization of biological specimen data in the field

**DOI:** 10.1002/ece3.4503

**Published:** 2018-09-17

**Authors:** Hasan Alhaddad, Bader H. Alhajeri

**Affiliations:** ^1^ Department of Biological Sciences Kuwait University Safat Kuwait

**Keywords:** animal sampling, biological specimen data, data management, fieldwork, images

## Abstract

Careful collection and organization of biological specimens and their associated data are at the core of field research (e.g., ecology, genetics). Fieldwork data are often collected by handwriting or unsystematically via an electronic device (e.g., laptop), a process that is time‐intensive, disorganized, and may lead to transcription errors, as data are copied to a more permanent repository. *SamplEase* is an iOS and Android application that is designed to ease the process of collecting biological specimen data in the field (data associated with biological samples, such as location, age, and sex). In addition to biological specimen data, *SamplEase* allows for the assignment of photographs to each collected sample, which provides visual records of each specimen in its environment. *SamplEase* outputs biological specimen data in a tabular format, facilitating subsequent analyses and dissemination. Despite the simplicity of *SamplEase*, no similar data management application is readily available for researchers.

## 1 INTRODUCTION

All research requires data. Data are often collected unmethodically and unsystematically. It is often the case that only after the completion of the field season, is this data organized and transcribed to a format that can be more readily archived, analyzed, and shared (i.e., a spreadsheet). This two‐step process of data collection is time‐intensive (reducing the time spent collecting samples) and reduces data quality (via omissions, transcription errors, etc.).

The usability of any dataset is directly related to its organization and formatting and the scientific value of the dataset increases as it is used by different research groups. Collaboration and data sharing across research groups are hindered by the use of disparate data collection and organization schemes—thus, a unified systematic data collection approach could increase collaboration and data sharing. While many researchers developed software to address and serve various scientific objectives (see lists in http://brunalab.org/apps/), none of these applications provide a simple solution to the collection of biological specimen data. The closest application to serve this purpose is *EpiCollect* (Aanensen, Huntley, Feil, al‐Own, & Spratt, 2009). However, the complex application design and multiple features, although valued, may limit its wide‐scale adoption and implementation across fields.

In designing *SamplEase*, we adopted the recommendations of Borer et al. concerning data formats, storage, and sharing potential as well as technical issues with naming files and categories of data (Borer, Seabloom, Jones, & Schildhauer, 2009). *SamplEase* is available in both Android and iOS platforms, designed to expedite collection, management, and sharing of biological specimen data in the field.

## 2 SAMPLEASE WORKFLOW

**Figure 1:**
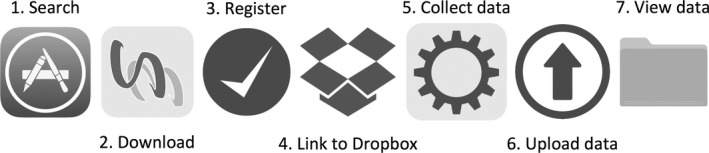
Overview of *SamplEase*. Steps one to four are conducted only once per session, while steps five to seven are conducted each time data are collected

The general idea behind developing *SamplEase* is to (1) rapidly and conveniently collect and store biological specimen data along with photographs for each biological sample, (2) export the data in a standardized table, formatted for easy accessibility and to ease sharing, and (3) instantly backup/deposit the tabulated data along with associated photographs to the researcher's personal file backup service (e.g., Dropbox). The typical workflow of *SamplEase* appears in Figure 1.

### 2.1 Search and download

*SamplEase* can be downloaded for free from either the App Store (iOS) or Google Play (Android).

### 2.2 Registration

**Figure 2:**
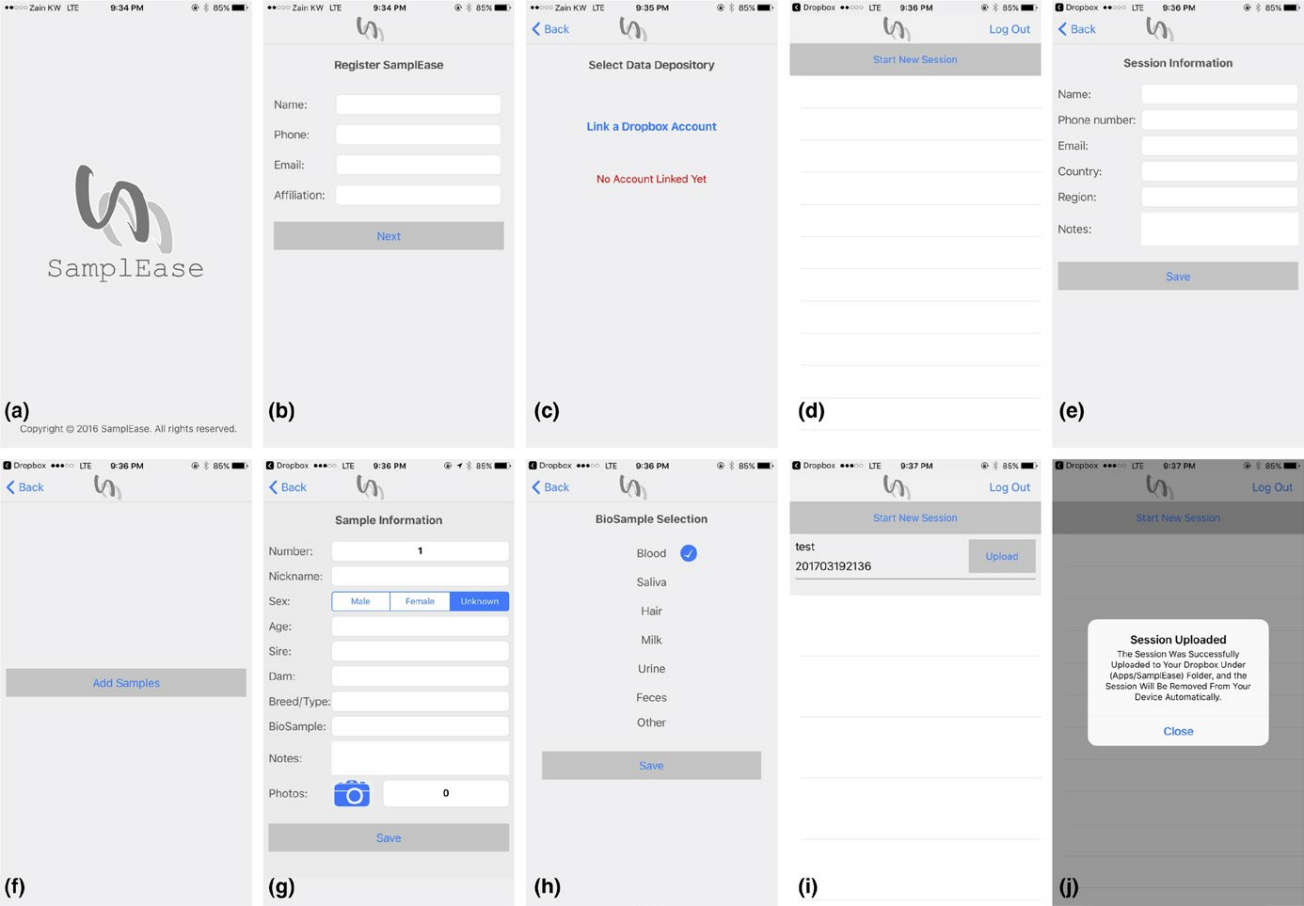
*SamplEase* screens (iOS version). (a) Opening screen. (b) Registration. (c) Link to Dropbox. (d) Start session. (e) Session information. (f) Sample addition. (g) Sample information. (h) Biosample selection. (i) Upload page. (j) Successful session upload

Registration is required before *SamplEase* (Figure 2a) can be used to collect data. Registration also ensures that the standardized table outputted by *SamplEase* includes the collector's basic information (name, phone number, email, and affiliation [Figure 2b]), which facilitates the sharing of biological samples. The user needs to register *SamplEase* only once, and after that, all collected data will include the collector's information.

### 2.3 Data repository

*SamplEase* needs to be linked to a data repository—this repository is used to automatically store/backup the data outputted from each *SamplEase* session. We chose a popular online file backup service, Dropbox (dropbox.com), to serve as the data repository for *SamplEase* data (Figure 2c). After the user links, his personal Dropbox account to *SamplEase*, a folder with the name “Apps” will appear in his Dropbox folder—*SamplEase* data (the table) and the associated photographs will be deposited in a subfolder entitled “*SamplEase”* (located inside the “Apps” folder). As in the aforementioned registration step (3), the user links *SamplEase* to his Dropbox account only once, and subsequently, all collected data will be automatically uploaded to the linked Dropbox account.

### 2.4 Session information

*SamplEase* allows for inputting session information (once per session), that is, then applied to all samples collected for this session. The session information includes (1) session name (e.g., breeder's name), (2) phone number (e.g., breeder's), (3) session email (e.g., breeder's), (4) country, (5) region, and (6) session notes. *SamplEase* automatically captures the date, time, and GIS coordinates, which are all included in the output table (Table 1).

**Table 1:** An example of a table outputted by *SamplEase* (transposed)

SamplEase fields	Entry type	Format	Example
Sample ID	Automatic	yyyymmddhhmm_#	201708012048_1
Date	Automatic	yyyymmddhhmm	201708012048
Nickname	Typed	Letters, numbers, symbols	Loli
Sex	List	Male, Female, Unknown	Male
Age	Typed	Letters, numbers, symbols	2 years
Sire	Typed	Letters, numbers, symbols	Sami
Dam	Typed	Letters, numbers, symbols	Tina
Breed	Typed	Letters, numbers, symbols	Racing breed
Biosample	List	Blood, Saliva, Hair, Milk, Urine, Feces, or Other	Hair
GIS location	Automatic	Latitude – Longitude	29.24436907 ‐ 48.07870861
Country	Typed	Letters, numbers, symbols	Kuwait
Region	Typed	Letters, numbers, symbols	Abdali
Sample notes	Typed	Letters, numbers, symbols	Subadult
Session name	Typed	Letters, numbers, symbols	Test
Session phone	Typed	Numbers, symbols	333‐333‐3333
Session email	Typed	Letters, numbers, symbols	breeder@email.com
Session notes	Typed	Letters, numbers, symbols	Collecting hair samples to start a camel biobank
First photograph	Automatic	yyyymmddhhmm_#_1	201708012048Test_1_1
Total photographs	Automatic	Number	2
Collector name	Typed	Letters, numbers, symbols	Researcher Richard
Collector phone	Typed	Numbers, symbols	555‐555‐5555
Collector email	Typed	Letters, numbers, symbols	camel@email.com
Collector affiliation	Typed	Letters, numbers, symbols	Kuwait University

### 2.5 Sample information

After the user completes inputting session information, *SamplEase* prompts the user to input data related to each sample, which is entered sequentially using the sample addition screen (Figure 2f). The first field in the sample information screen is (an automatically generated) sequential sample number (Figure 2g). Sample number is later concatenated with session information to generate a unique sample identification number, which is included in the output table.

### 2.6 SamplEase output

After the completion of a sampling session, the data are uploaded to the linked Dropbox account as a compressed file (.zip), which is deposited in a folder with the path: “Dropbox/Apps/SamplEase.” The .zip file is given a unique name based on the collection date and time, and the name of the sampling session, in the following format: “year‐month‐day‐hour‐minute‐session name.zip” (or yyyymmddhhmmSessionName.zip). The name of the (.zip) file, which begins with the date and time in reverse chronological order, ensures that the files are automatically sorted based on collection time, with older samples listed on top of newer ones. The relatively small size of the (.zip) file facilitates data sharing, as well as long‐term data archiving in Dropbox and/or a physical hard drive.

Uncompressing the (.zip) file on a personal computer leads to a folder that includes *SamplEase* output files: (a) a comma delimited (.csv) file that contains the biological specimen data in the form of a table, and (b) all the photographs captured during the sampling session, in a Portable Network Graphics format (.png; each image = ~1.5 MB on an iPhone 7). The (.csv) table has the same name as the folder that houses it, and contains the data entered in the sampling session arranged into 23 columns (Table 1).

The names of the photographs follow the same scheme (see above), where each image is identified by the date, time, and session name, followed by the sample number, and finally the photograph number (e.g., the third photograph of the second sample in a particular session would have the following name: yyyymmddhhmmSessionName_2_3.png). This systematic naming scheme provides easy access to the images, especially when many samples are collected in a sampling session, with many photographs taken for each sample.

Photographing individuals from which samples are taken ensures that phenotype can be linked with genotype. More specifically, it allows the researcher to spend less time collecting phenotypic data in the field, and more time collecting biological samples, as the phenotypic characterization of the sampled individuals can be done off‐site (i.e., from the images).

For a video walkthrough of a typical *SamplEase* work session, along with the associated output (.zip) file, see Videos S1 and S2 and Data S1.

## 3 *SAMPLEASE* LIMITATIONS

*SamplEase* has a number of limitations that are subject to future improvements: (a) the application is limited to one online file backup service (Dropbox) and (b) the photographs have a relatively large file size, which may quickly fill up the allocated cloud storage space.

## 4 *SAMPLEASE* AND CAMEL RESEARCH

As an illustration of the utility of *SamplEase* in biological research, we present an outline of our ongoing research on dromedary camels. Our investigations focus on the molecular, biochemical, and morphological variations, which requires the collection of both biological samples and phenotypic data for each sample. *SamplEase* eased the process of collecting biological specimens (i.e., blood, saliva, tail‐hair, milk etc.) of our sampled camels and their accompanying specimen data.

**Figure 3:**
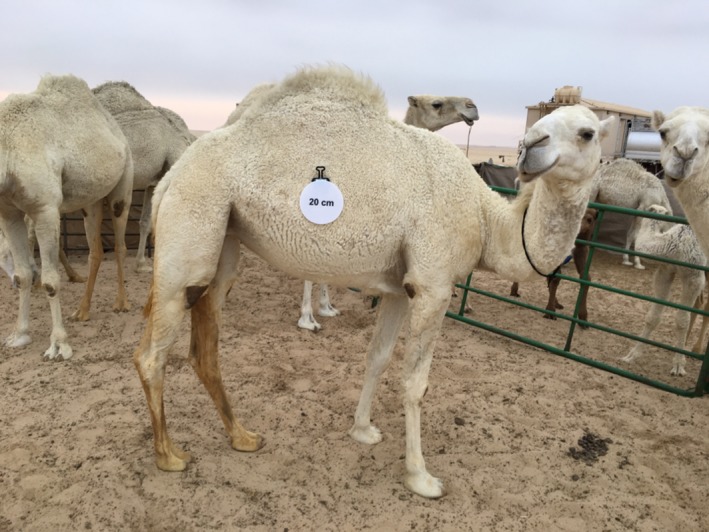
Example of a photograph taken using *SamplEase* of a white camel

**Figure 4:**
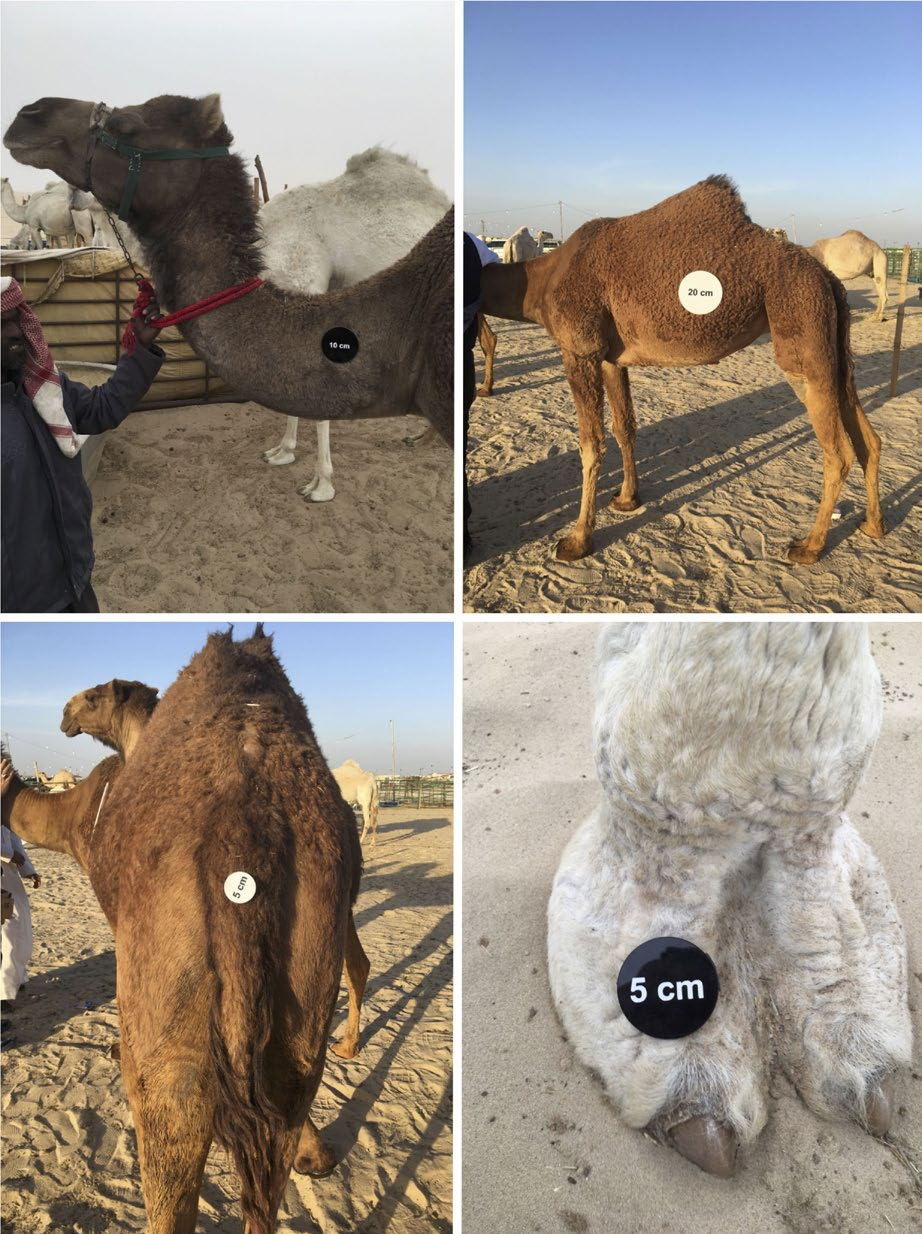
Images of camel neck, body, tail, and foot captured with *SamplEase*. Scale disks (5, 10, 20 cm) are placed on the body parts to allow for extraction of scale factors

**Figure 5:**
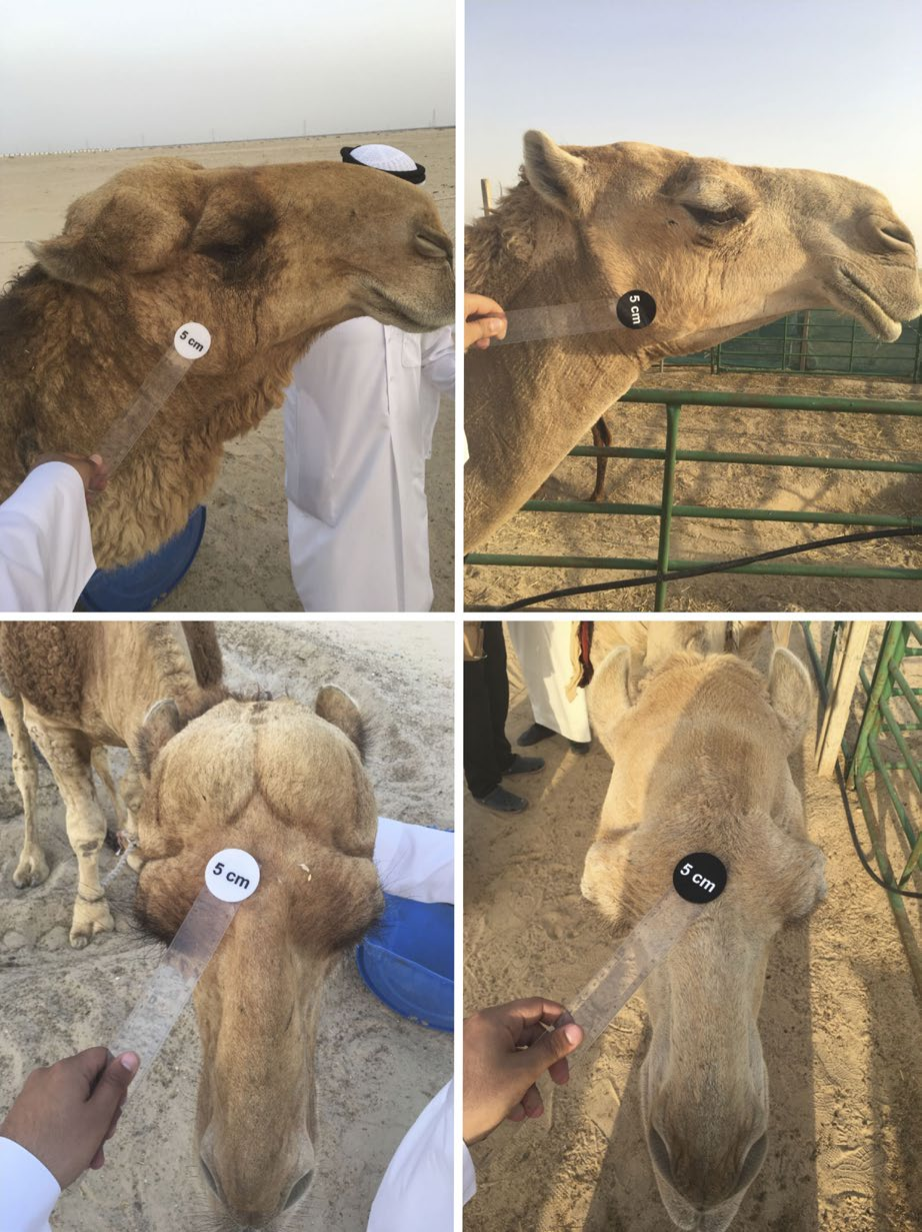
Lateral and dorsal views of camel heads. A 5 cm scale was placed on each image for morphometric analyses

We have also used *SamplEase* images (Figure 3) to extract additional detailed morphological information and health status for each sampled camel. This information, along with the biological specimens, is intended for use in investigating the genetic basis of various morphological attributes. We are also experimenting with the use of *SamplEase* images to explore morphometric variation in camels (Figures 4 and 5).

## 5 CONCLUSION

*SamplEase* is an improvement over commonly used methods of collecting biological samples and their associated biological specimen data information in the field, by allowing for systematic and rapid entry of descriptive and photographic data for each collected DNA sample. Moreover, the format in which the data are outputted from *SamplEase* is simple and organized, simplifying subsequent data archiving, sharing, and analysis. The automatic upload of biological specimen data to the linked Dropbox account mitigates information loss, and provides a convenient medium for data storage and sharing. *SamplEase*’ simple design makes it flexible, allowing it to be used by researchers from different fields.

## CONFLICT OF INTEREST

None declared.

## AUTHOR'S CONTRIBUTIONS

HA and BHA conceived and designed the application, and wrote the manuscript.

## DATA ACCESSIBILITY

The application is available for free for iOS (App Store) and Android (Google Play).

## Supporting information


** **

** **

** **

** **

** **

** **
