# Supplementary figures and images for: SamplEase: a simple application for collection and organization of biological specimen data in the field
Source: Ecol Evol. 2018 Sep 17;8(20):10266–71. doi: 10.1002/ece3.4503 (PMC6206197; doi:10.1002/ece3.4503)

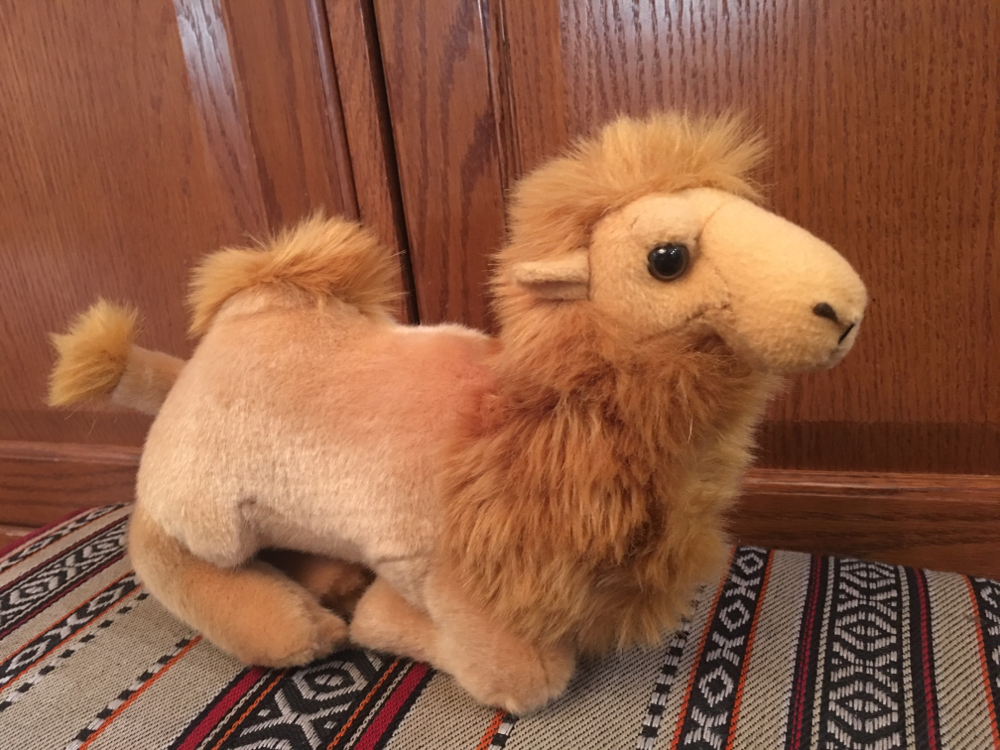

Supplement: Supplementary file 1 [file ECE3-8-10266-s001.png]

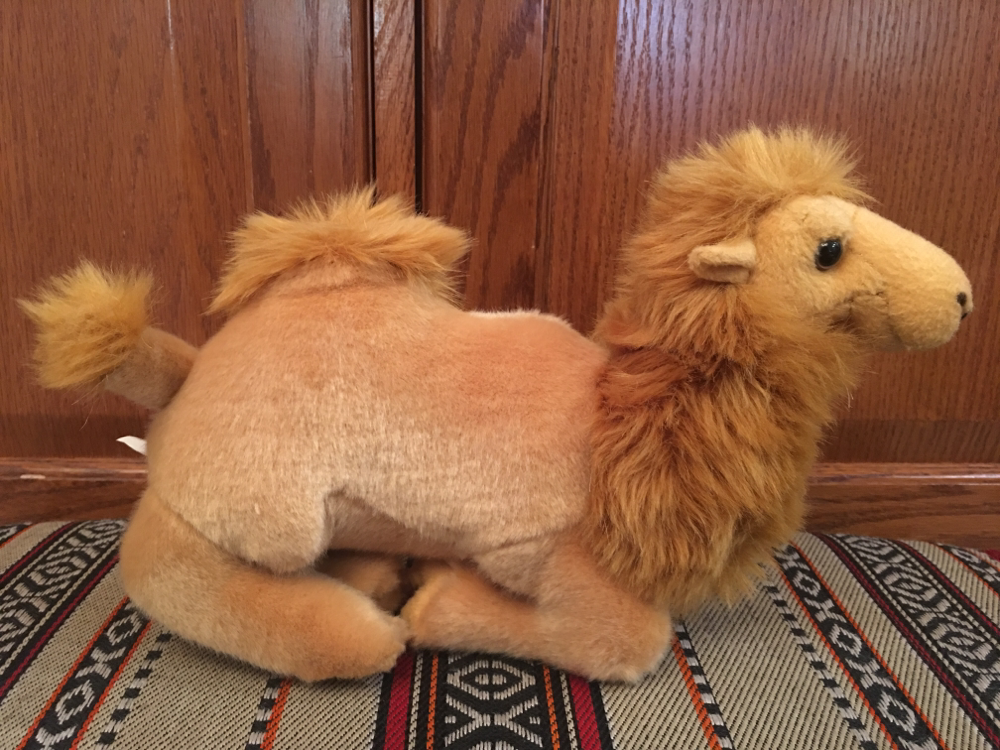

Supplement: Supplementary file 2 [file ECE3-8-10266-s002.png]

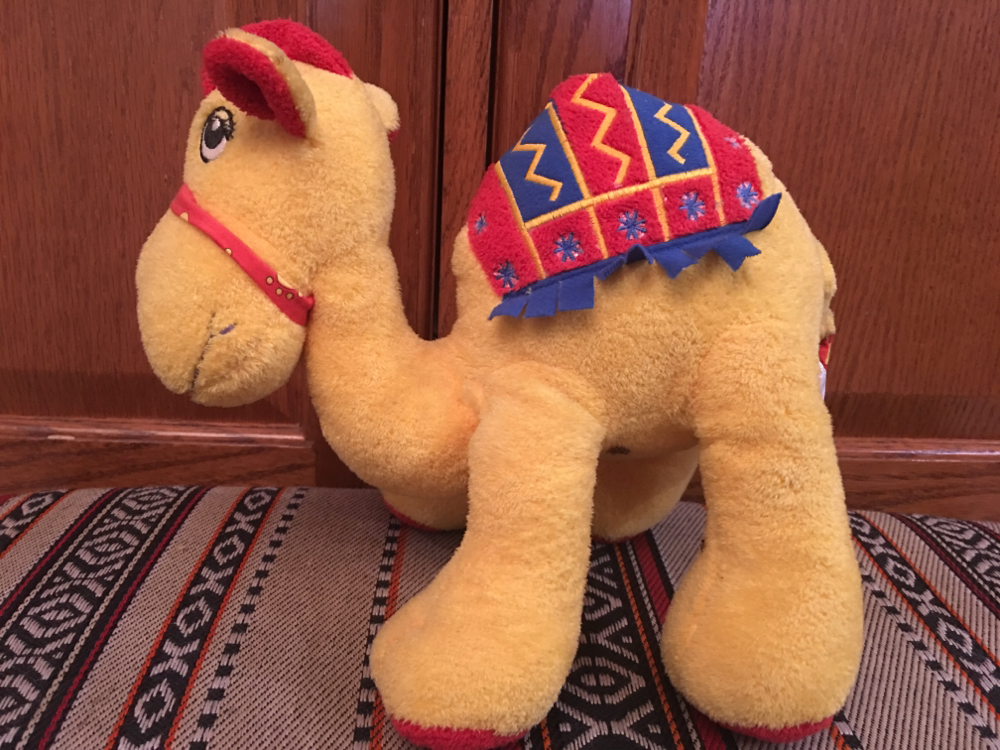

Supplement: Supplementary file 3 [file ECE3-8-10266-s003.png]

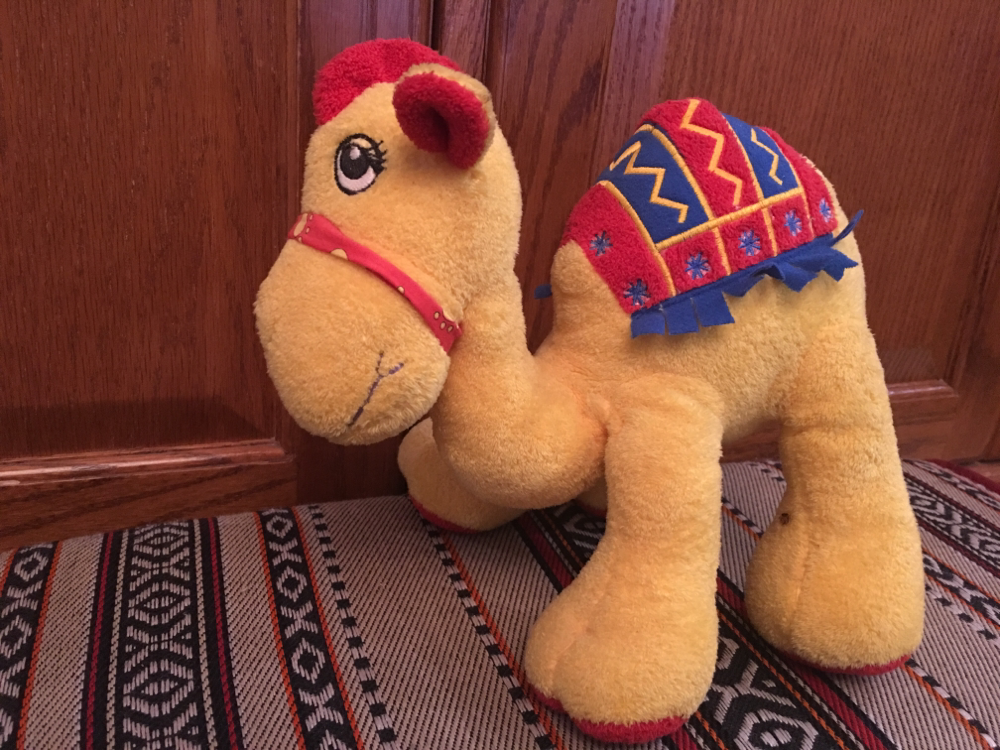

Supplement: Supplementary file 4 [file ECE3-8-10266-s004.png]
